# Calcified Nodules in Non-Culprit Lesions with Acute Coronary Syndrome Patients

**DOI:** 10.31083/j.rcm2504136

**Published:** 2024-04-07

**Authors:** Xi Wu, Mingxing Wu, Haobo Huang, Lei Wang, Zhe Liu, Jie Cai, He Huang

**Affiliations:** ^1^Department of Cardiology, Xiangtan Central Hospital, 411100 Xiangtan, Hunan, China

**Keywords:** calcified nodule, acute coronary syndrome, intravascular ultrasound atherosclerosis

## Abstract

**Background:**

Calcified nodules (CN) have been linked to unfavorable clinical outcomes. However, there is a lack of systematic studies on non-culprit lesions with CN in patients with acute coronary syndromes (ACS). This study aims to investigate the frequency, distribution, predictors, and outcomes of CN in non-culprit lesions among ACS patients.

**Methods:**

We included 376 ACS patients who received successful stent placement in their culprit lesions. Intravascular ultrasound (IVUS) was performed to evaluate non-culprit lesions in left main arteries and all three coronary arteries (CA). CN was defined as accumulations of small nodular calcium deposits exhibiting a convex shape protruding into the lumen.

**Results:**

CNs was identified in 16.9% (121 of 712) per artery and 26.9% (101 of 376) per patient. They were predominantly located at the mid portion of the right coronary artery (26.3%) and the bifurcation site (59.9%). Patients with CN were older (63.57 ± 8.43 vs. 57.98 ± 7.15, *p* < 0.001) and had a higher prevalence of diabetes mellitus (55.4% vs. 42.2%, *p* = 0.022). However, there were no significant differences in baseline characteristics observed after propensity score matching (PSM). Multivariate analysis revealed that CN were independently associated with major adverse cardiovascular events (MACE) both before and after PSM (hazard ratio (HR): 0.341, 95% confidence interval (95% CI): 0.140–0.829, *p* = 0.018; HR: 0.275, 95% CI: 0.108–0.703, *p* = 0.007, respectively). During the observational period of 19.35 ± 10.59 months, the occurrence of MACE was significantly lower in patients with CN before and after PSM (5.9% vs. 16.7%, *p* = 0.046; 4.0% vs. 18.1%, *p* = 0.011; respectively).

**Conclusions:**

CN in non-culprit lesions with ACS patients was prevalent and caused fewer adverse clinical outcomes.

## 1. Introduction

Pathologically, calcified nodules (CN) are characterized by a disruption of the fibrous cap, the presence of fibrin or platelet-rich thrombus, and the accumulation of eruptive, dense, CN that penetrate the luminal surface. Compared to plaque rupture (PR) and plaque erosion (PE), CN are considered to be a less common cause of acute coronary syndromes (ACS) [1, 2]. Intravascular ultrasound (IVUS) is a highly sensitive and specific imaging modality that provides detailed qualitative and quantitative information on underlying plaque morphology, including the detection of coronary calcium. Given the potential impact of lesion calcification with this unique morphology on clinical outcomes, recent studies have associated CN with adverse events following percutaneous coronary intervention (PCI), such as target lesion revascularization (TLR) [3, 4, 5] and refractory in-stent restenosis (IRS) [6]. However, the morphological features and clinical consequences of CN in non-culprit lesions in ACS patients are unknown. The goals of this *in-vivo* IVUS study are to establish the frequency, distribution, angiographic and intravascular ultrasound (IVUS) presentation, predictors, and outcomes of CN in ACS patients with non-culprit lesions.

## 2. Materials & Methods

### 2.1 Research Population

**Fig. 1.**
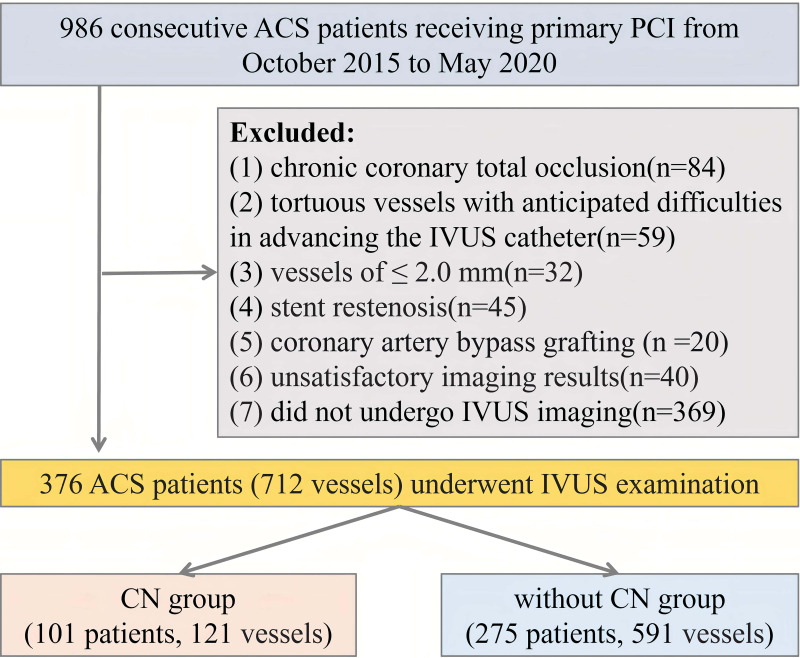
**The study flow chart**. Abbreviations: ACS, acute coronary syndrome; CN, calcified nodule; PCI, percutaneous coronary intervention; IVUS, intravascular ultrasound.

The study group included 986 patients with ACS who had primary PCI between October 2015 and May 2020. ST-segment elevation myocardial infarction (STEMI) and non-ST-segment elevation myocardial infarction (NSTEMI) were both classified as ACS [7]. In addition, cases of unstable angina pectoris (uAP) without cardiac enzyme elevation were included. The presence of a culprit lesion was diagnosed based on angiographic morphology, electrocardiogram (ECG) data, and anomalies in left ventricular wall motion. A culprit lesion was more likely to be associated with more severe stenoses and evidence of recent plaque disruption, with a filling defect on angiography suggestive of thrombus. Lesions with a visible diameter stenosis of more than 30% are considered to be non-culprit. Among the 986 patients, exclusion criteria were as follows: (1) chronic total occlusion of coronary arteries (CA); (2) tortuous vessels that would pose difficulties in advancing the IVUS catheter; (3) vessel diameter ≤2.0 mm; (4) stent restenosis; (5) previous coronary artery bypass grafting (CABG); (6) unsatisfactory imaging results; and (7) lack of IVUS imaging (Fig. 1). Conducted under the ethical tenets of the Declaration of Helsinki, the study secured institutional ethics committee endorsement. All participants provided written, informed consent for the PCI interventions.

### 2.2 PCI Procedures and Clinical Follow-up

Following the successful stenting of all culprit lesions, IVUS was conducted to evaluate non-culprit lesions in the left main coronary arteries (LMCA) and all three coronary arteries. Procedural decisions were made based on the individual PCI operator’s judgment and discretion. Dual antiplatelet therapy was maintained for not less than one year after PCI. Physicians collected relevant data through new hospital admissions, telephone conversations, or clinic appointments subsequent to the PCI.

### 2.3 Quantitative Coronary Angiography (QCA) Analysis 

Quantitative coronary angiography (QCA) analysis was performed on the non-culprit lesion using QAngio software (v2.1.9, Medis, Leiden, the Netherlands) for offline analysis. The QCA study took into account numerous factors, including minimal lumen diameter, lesion length, reference vessel diameter, angiographic calcium (moderate or severe) and angiographic haziness [8, 9]. Based on the American Heart Association classification, the three epicardial arteries were divided into different segments, which included the left main (LM) artery segment (5), proximal segments (1, 6, 11), mid segments (2, 7, 13), and distal segments (3, 4, 8–10, 12, 14, 15) [10].

### 2.4 IVUS Image Analysis 

**Fig. 2.**
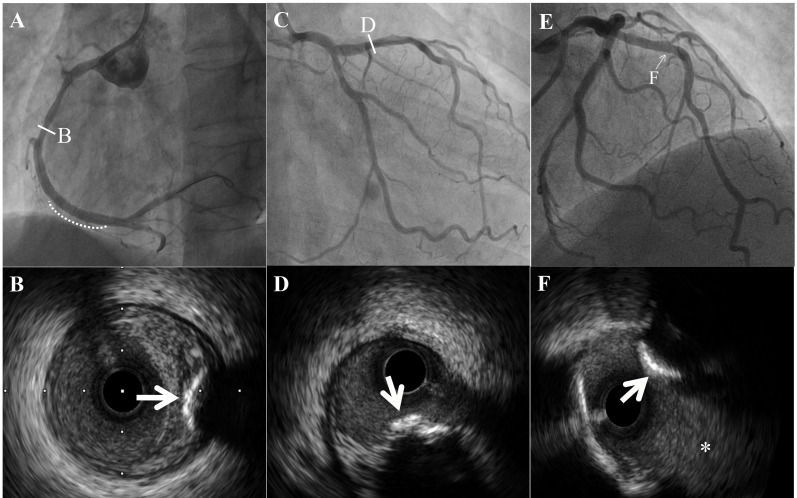
**Intravascular ultrasound images of a calcified nodule (CN)**. (A) White line indicate CN at the mid of right coronary artery. White dotted line indicate stent at culprit lesion. (B) Calcified nodule had a convex and irregular surface (white arrow), between intima and external elastic membrane. (C) White line indicate CN at the proximal of left anterior descending artery. (D) CN was superficial of the intima (white arrow). (E) White arrowhead indicate CN at the bifurcation site and the distal site of the branch. (F) CN was superficial of the intima (white arrow). White asterisk indicate the diagonal branch.

IVUS images were acquired using a 40-MHz OptiCross™ catheter (Boston Scientific, Marlborough, MA, USA) within all three epicardial arteries. After intracoronary administration of nitroglycerin (100–200 µg; H44020569, Guangzhou Baiyun Mountain Mingxing pharmaceutical Company, Guangzhou, China), IVUS was automatically pulled back at 0.5 mm/s from distal to proximal references. CN was defined as a convex surface, protruding calcification from luminal surface [11]. For each subject, CN were classified as either single (occurring 1 solitary CN in one patient only) or multiple (occurring in a single vessel with more than or equal to two nodules or in at least two vessels with one nodule each). Following CN identification, the proximal and distal reference segments representing the most normal-looking cross sections within 10 mm of the nodule were chosen for additional research (Fig. 2). The slice with the narrowest lumen and the highest plaque burden (PB) was chosen as the minimal lumenal area (MLA) site among these reference segments. For quantitative investigation, the cross-sectional areas (CSA) of the external elastic membrane (EEM), lumen, and plaque plus media at the CN, MLA site, and proximal and distal reference segments were assessed. PB was estimated by multiplying the plaque plus media CSA by 100 and then dividing by the EEM CSA. At the CN and MLA sites, the remodeling index was calculated by dividing the EEM by the average EEM of the proximal and distal reference segments. Lumen area stenosis was calculated by 1 minus MLA divided by the average reference lumen CSA at the calcified nodule site and at the MLA site. Calcium analysis entailed detecting the location of calcium and measuring the maximum arc of calcium. Volumes were calculated using Simpson’s rule. According to the published research, the quantitative IVUS analysis included calcium surface (smooth or irregular), visible tissue between the lumen and calcium (absent or present), eccentricity of plaque (concentric or eccentric) and calcified nodule surface echogenicity (isoechoic, or hyperechoic) [11]. Hyperechoic tissue measured as echogenicity is brighter than the reference vessel adventitia with shadowing [12]. The CN identification and quantitative analyses were carried out independently by two cardiologists who were blinded to the clinical presentation. For the diagnosis of CN and IVUS image quantitative analyses, the intra-observer and inter-observer variability demonstrated good agreement by 2 independent cardiologists (XW and HH) who blinded to the clinical presentation, coronary angiographic, and laboratory data (κ = 0.92 and 0.89, respectively).

### 2.5 Outcomes

The study’s primary end point was the appearance of major adverse cardiovascular events (MACE) in non-culprit lesions. MACEs included cardiac-related mortality, the development of recurrent ACS attributable to progression of non-culprit lesions, or hospital readmission due to unstable or worsening angina. Secondary endpoints included the existence of each MACE component.

### 2.6 Statistical Analysis

Statistical analyses were conducted using SPSS 22.0 (IBM Corporation, Armonk, NY, USA). Continuous variables were presented as the mean ± standard deviation (SD) or medians and interquartile ranges (IQRs). Comparisons between groups were performed using the *t*-test. Categorical variables were compared using the Chi-square test. Survival curves were estimated using the Kaplan-Meier method. The log rank test was used to assess differences between patients with and without CN. The propensity score was estimated using logistic regression models, with MACE as the outcome and baseline clinical demographics and anatomic characteristics as predictors. Patients with and without CN were matched by propensity score matching (PSM) on a 1:1 basis using the nearest neighbor matching algorithm, with a caliper width equal to 0.01 of the standard deviation of the propensity score. Intra-observer and inter-observer variability for the diagnosis of a calcium nodule was measured by the test of concordance. A *p* value < 0.05 was considered statistically significant.

## 3. Results

### 3.1 Baseline Characteristics of Patients with and without CN

From October 2015 to May 2020, among 986 ACS patients undergoing PCI in our hospital, a total of 376 ACS patients (101 in the CN group and 275 in the without CN group) were included in the study population. Patients with CN were older and had a higher prevalence of diabetes mellitus (DM). There was no significant difference in the incidence of male gender, past myocardial infarction, hypertension, smoking, prior PCI, familial CA disease, chronic kidney disease (CKD), hemodialysis, and the presentation of ACS, between the two groups. The laboratory data and medical therapies at discharge were also similar between the two groups (Table 1). After PSM, the baseline characteristics of the two groups were well-balanced in the analysis (Table 2).

**Table 1.** **Baseline clinical characteristics**.

Variables		with CN (n = 101)	without CN (n = 275)	*p* value
Age (years)		63.57 ± 8.43	57.98 ± 7.15	<0.001
Female, n (%)		62 (61.4)	178 (64.7)	0.550
Hypertension, n (%)		73 (72.3)	206 (74.9)	0.605
Diabetes, n (%)		56 (55.4)	116 (42.2)	0.022
Current smokers, n (%)		30 (29.7)	74 (26.9)	0.591
Prior myocardial infarction, n (%)		19 (18.8)	55 (20.0)	0.797
Family history of CAD, n (%)		21 (20.8)	54 (19.6)	0.804
History of dyslipidemia, n (%)		38 (37.6)	104 (37.8)	0.973
LVEF (%)		61.94 ± 6.94	63.01 ± 7.51	0.213
BMI, ${\mathrm{kg/m}}^{2}$, (IQR)		24.27 (22.83–26.38)	24.33 (22.84–25.72)	0.482
CKD (eGFR <60), n (%)		26 (25.7)	72 (26.2)	0.931
Hemodialysis, n (%)		8 (7.9)	22 (8.0)	0.980
Multivessel disease, n (%)		45 (44.6)	118 (42.9)	0.775
A history of PCI, n (%)		15 (14.9)	44 (16.0)	0.786
ACS presentation, n (%)				0.659
	STEMI	12 (11.9)	43 (15.6)	
	NSTEMI	59 (58.4)	154 (56.0)	
	UAP	30 (29.7)	78 (28.4)	
Calcium (mg/dL)		9.18 ± 0.67	9.11 ± 0.63	0.420
Phosphorus (mg/dL)		3.61 ± 0.83	3.52 ± 0.74	0.309
ALP (U/L)		230.5 ± 88.52	226.5 ± 119.5	0.759
Hemoglobin A1c (%), (IQR)		7.43 (6.47–8.33)	7.27 (6.48–8.25)	0.586
Triglycerides (mmol/L)		1.41 ± 0.97	1.55 ± 1.27	0.313
Total cholesterol (mmol/L)		4.32 ± 1.23	4.04 ± 1.31	0.062
HDL-C (mmol/L)		1.09 ± 0.39	1.15 ± 0.45	0.240
LDL-C (mmol/L)		2.91 ± 0.79	2.83 ± 0.91	0.436
hs-CRP (mg/L)		8.41 ± 4.98	8.19 ± 3.69	0.643
Baseline TNT (ng/mL)		3.54 ± 2.21	3.32 ± 1.67	0.280
Baseline CK-MB (IU/L)		94.53 ± 60.08	92.99 ± 69.21	0.843
Medical therapies at discharge, n (%)				
	Aspirin	101 (100)	275 (100)	>0.999
	P2Y12 inhibitor	101 (100)	275 (100)	>0.999
	ACEI or ARB	68 (67.3)	192 (69.8)	0.643
	Beta-blocker	30 (29.7)	62 (22.5)	0.152
	Statin	78 (77.2)	201 (73.1)	0.416
	Nitrate	70 (69.3)	187 (68.0)	0.809

Note: values are mean ± SD, n (%) or median (interquartile range). Abbreviations: STEMI, ST-segment elevation myocardial infarction; NSTEMI, non-ST-segment elevation myocardial infarction; LVEF, left ventricular ejection fraction; BMI, body mass index; CAD, coronary artery disease; LDL-C, low-density lipoprotein cholesterol; HDL-C, high-density lipoprotein cholesterol; hs-CRP, high sensitivity C-reactive protein; TNT, troponin-T; CK-MB, creatine kinase MB; ARB, angiotensin receptor blockers; ACEI, angiotensin-converting enzyme inhibitors; CKD, chronic kidney disease; ALP, alkaline phosphatase; CN, calcified nodule; PCI, percutaneous coronary intervention; UAP, unstable angina pectoris; ACS, acute coronary syndrome; eGFR, estimated glomerular filtration rate; IQR, interquartile range.

**Table 2.** **Baseline clinical characteristics after PSM**.

Variables		with CN (n = 101)	without CN (n = 83)	*p* value
Age (years)		63.57 ± 8.43	62.06 ± 7.02	0.193
Female, n (%)		54 (60.7)	54 (60.7)	>0.999
Hypertension, n (%)		61 (68.5)	63 (70.8)	0.872
Diabetes, n (%)		45 (50.6)	40 (44.9)	0.061
Current smokers, n (%)		26 (29.2)	28 (31.5)	0.702
Prior myocardial infarction, n (%)		15 (16.9)	15 (16.9)	>0.999
Family history of CAD, n (%)		16 (18.0)	17 (19.1)	0.802
History of dyslipidemia, n (%)		29 (32.6)	33 (37.1)	0.721
LVEF (%)		62.94 ± 6.94	64.3 ± 7.01	0.188
BMI, ${\mathrm{kg/m}}^{2}$ (IQR)		24.52 ± 2.51	24.35 ± 2.11	0.626
CKD (eGFR <60), n (%)		23 (25.8)	22 (24.7)	0.792
Hemodialysis, n (%)		5 (5.6)	5 (5.6)	>0.999
Multivessel disease, n (%)		40 (44.9)	42 (47.2)	0.705
A history of PCI, n (%)		12 (13.5)	10 (11.2)	0.686
ACS presentation				0.875
	STEMI, n (%)	10 (11.2)	9 (10.1)	
	NSTEMI, n (%)	54 (60.7)	53 (59.6)	
	UAP, n (%)	25 (28.1)	27 (30.3)	
Calcium (mg/dL)		9.18 ± 0.67	9.15 ± 0.61	0.812
Phosphorus (mg/dL)		3.61 ± 0.83	3.55 ± 0.78	0.665
ALP (U/L)		230.5 ± 88.52	238.02 ± 138.75	0.656
Hemoglobin A1c (%) (IQR)		7.43 (6.47–8.32)	7.29 (6.32–8.46)	0.854
Triglycerides (mmol/L)		1.41 ± 0.97	1.61 ± 1.31	0.253
Total cholesterol (mmol/L)		4.32 ± 1.23	3.99 ± 1.36	0.078
HDL-C (mmol/L)		1.09 ± 0.39	1.11 ± 0.49	0.728
LDL-C (mmol/L)		2.91 ± 0.79	2.83 ± 0.96	0.557
hs-CRP (mg/L)		8.41 ± 4.98	8.74 ± 3.61	0.617
Baseline TNT (ng/mL)		3.54 ± 2.21	3.15 ± 1.55	0.171
Baseline CK-MB (IU/L)		94.53 ± 60.08	91.91 ± 69.98	0.784
Medical therapies at discharge , n (%)				
	Aspirin	89 (100)	89 (100)	>0.999
	P2Y12 inhibitor	89 (100)	89 (100)	>0.999
	ACEI or ARB	61 (68.5)	63 (70.8)	0.872
	Beta-blocker	24 (27.0)	25 (28.1)	0.839
	Statin	67 (75.3)	64 (71.9)	0.459
	Nitrate	62 (69.7)	63 (70.8)	0.903

Note: values are mean ± SD, n (%) or median (interquartile range). Abbreviations: PSM, propensity score matching; LVEF, left ventricular ejection fraction; BMI, body mass index; CAD, coronary artery disease; LDL-C, low-density lipoprotein cholesterol; HDL-C, high-density lipoprotein cholesterol; hs-CRP, high sensitivity C-reactive protein; TNT, troponin-T; CK-MB, creatine kinase MB; ARB, angiotensin receptor blockers; ACEI, angiotensin-converting enzyme inhibitors; CKD, chronic kidney disease; ALP, alkaline phosphatase; CN, calcified nodule; PCI, percutaneous coronary intervention; STEMI, ST-segment elevation myocardial infarction; NSTEMI, non-ST-segment elevation myocardial infarction; UAP, unstable angina pectoris; ACS, acute coronary syndrome; eGFR, estimated glomerular filtration rate; IQR, interquartile range.

### 3.2 Characteristics of CN

Among the 376 patients with ACS who underwent imaging of 712 vessels for analysis (48 left main, 252 left anterior descending [LAD], 186 left circumflex coronary artery [LCx], and 226 right CA [RCA]), a total of 137 CN were detected in 121 vessels in 101 patients. The prevalence of CN was 16.9% per artery (121 out of 712) and 26.9% per patient (101 out of 376). Specifically, there were 47 nodules in the LAD in 38 patients, 36 nodules in the LCx in 27 patients, and 54 nodules in the RCA of 36 patients. Notably, no CN were observed in the LMCA. Overall, 16.7% of LAD vessels (42 out of 252), 19.9% of LCx vessels (34 out of 186), and 22.1% of RCA vessels (50 out of 226) contained only one CN. Multiple CN were found in 11 CA (1.5%) among 9 patients (2.4%). Specifically, 2.0% of LADs (5 out of 252), 1.1% of LCxs (2 out of 186), and 1.8% of RCAs (4 out of 226) exhibited multiple nodules. Only 4 CN (2.9%) showed evidence of moderate calcium on angiography, 1 (0.7%) exhibited severe calcium, and 5 (3.6%) appeared hazy on angiography. The remaining 127 CN (92.7%) appeared normal on angiography. The number of coronary calcified nodules per patient was 1.4. The average volume of calcified nodules for per vessel and per patient are 1.2 ${\mathrm{mm}}^{3}$ (0.37–2.92) and 1.2 ${\mathrm{mm}}^{3}$ (0.41–2.98). 87.6% (120 out of 137) of calcified nodules had irregular calcium. 55.5% (76 out of 137) of calcified nodules exhibited isoechoic echogenicity followed by hyperechoic echogenicity (44.5%). Visible tissue between the lumen and calcium accounted for 67.9% (93 out of 137) of calcified nodules. 85.4% (117 out of 137) of calcified plaques had an eccentric shape.

### 3.3 IVUS Findings of CN

**Fig. 3.**
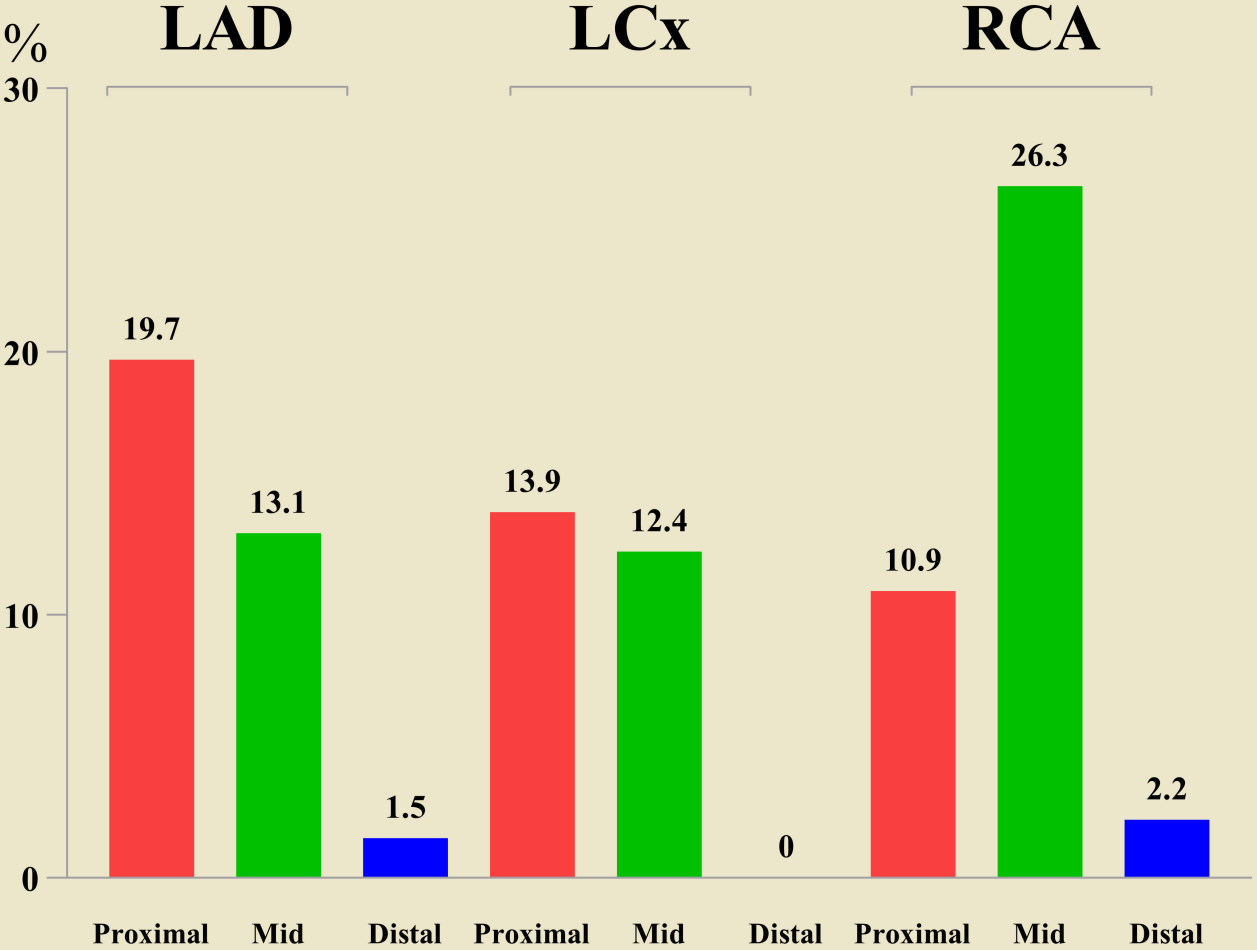
**Distribution of calcified nodule in the coronary arteries**. Abbreviations: LAD, left anterior descending coronary artery; LCx, left circumflex coronary artery; RCA, right coronary artery.

The CSA of the lumen at the site of the CN was significantly larger compared to the MLA site (8.53 ${\mathrm{mm}}^{2}$ [IQR, 8.27–8.81 ${\mathrm{mm}}^{2}$] vs. 7.29 ${\mathrm{mm}}^{2}$ [IQR, 6.89–7.75 ${\mathrm{mm}}^{2}$]; *p* < 0.001). Additionally, the CSA of plaque plus media and the PB were correspondingly smaller at the CN site (7.54 ${\mathrm{mm}}^{2}$ [IQR, 7.32–7.80 ${\mathrm{mm}}^{2}$] vs. 8.89 ${\mathrm{mm}}^{2}$ [IQR, 8.46–9.20 ${\mathrm{mm}}^{2}$]; *p* < 0.001; 44.80% [IQR, 43.10–46.01%] vs. 56.41% [IQR, 54.55–57.96%]; *p* < 0.001, respectively) (Table 3). The CN were distributed as follows: 40.1% (55 out of 137) were distal to the MLA site, 45.3% (62 out of 137) were proximal to the MLA site, and 14.6% (20 out of 137) were at the MLA site. The CN were most commonly seen in the mid-portion of RCA, followed by the proximal region of the LAD artery (Fig. 3). The arc of the CN measured 29.3° (IQR, 26.3–32.1), with 90.5% (124 out of 137) located superficially in the intima and 9.5% (13 out of 137) in a mixed position between the intima and external elastic membrane. Among the CN, 59.9% (82 out of 137) were distributed at the bifurcation site, with 34 nodules located at the proximal site of the branch and 48 nodules at the distal site. The average distance between the CN site and the branch was 1.6 mm (IQR, 1.3–2.0). Tables 4,5 show the coronary angiographic and IVUS findings along the whole length of the three imaging CA in patients with and without CN.

**Table 3.** **IVUS measures of calcified nodules site and the minimum lumen area site**.

Variables	Calcified nodules site (n = 137)	MLA site (n = 137)	*p* value
EEM CSA, ${\mathrm{mm}}^{2}$ (IQR)	15.53 (14.66–16.45)	15.33 (14.48–16.46)	0.467
Lumen CSA, ${\mathrm{mm}}^{2}$ (IQR)	8.53 (8.27–8.81)	7.29 (6.89–7.75)	<0.001
Plaque plus media CSA, ${\mathrm{mm}}^{2}$ (IQR)	7.54 (7.32–7.80)	8.89 (8.46–9.20)	<0.001
Plaque burden, %	44.80 (43.10–46.01)	56.41 (54.55–57.96)	<0.001
Remodeling index (IQR)	0.97 (0.96–0.98)	0.98 (0.97–0.99)	0.132

Abbreviations: MLA, minimum lumen cross-sectional area; EEM, external elastic membrane; CSA, cross-sectional area; IVUS, intravascular ultrasound; IQR, interquartile range.

**Table 4.** **Coronary angiographic and IVUS findings of patients with calcified nodule and without calcified nodule**.

Variables	Calcified nodules (n = 101)	Without calcified nodules (n = 275)	*p* value
Total length analyzed, mm (IQR)	184.43 (170.81–199.08)	187.13 (171.45–202.22)	0.371
Nonculprit lesions, n	5.0 (4.0–6.0)	5.0 (4.0–6.0)	0.276
Length of nonculprit lesions, mm (IQR)	43.96 (34.53–55.22)	45.26 (31.09–62.12)	0.290
Average EEM CSA, ${\mathrm{mm}}^{2}$ (IQR)	15.69 (13.13–17.58)	15.43 (12.89–17.83)	0.929
Average lumen CSA, ${\mathrm{mm}}^{2}$ (IQR)	7.91 (7.34–8.52)	7.95 (7.09–9.01)	0.118
Average P+M CSA, ${\mathrm{mm}}^{2}$ (IQR)	8.72 (7.97–9.65)	9.04 (8.31–9.73)	0.084
Plaque burden, %	47.76 (44.43–50.12)	47.43 (43.89–50.51)	0.339
Reference diameter, mm	3.10 ± 0.52	3.13 ± 0.51	0.236
MLD, mm	2.64 ± 1.44	2.58 ± 1.59	0.282
MLA, ${\mathrm{mm}}^{2}$	2.81 ± 0.64	2.89 ± 0.60	0.233
Reference area, ${\mathrm{mm}}^{2}$	13.94 ± 4.61	14.32 ± 5.12	0.480

Note: values are mean ± standard deviation (SD) or median (interquartile range). Abbreviations: EEM, external elastic membrane; CSA, crosssectional area; P+M, plaque+media; IVUS, intravascular ultrasound; MLD, minimum lumen diameter; MLA, minimal lumenal area; IQR, interquartile range.

**Table 5.** **Coronary angiographic and IVUS findings of patients with calcified nodule and without calcified nodule after PSM**.

Variables	Calcified nodules (n = 101)	Without calcified nodules (n = 83)	*p* value
Total length analyzed, mm (IQR)	184.43 (170.81–199.08)	187.64 (173.08–200.88)	0.129
Nonculprit lesions, n	5.0 (4.0–6.0)	5.0 (4.0–6.0)	0.072
Length of nonculprit lesions, mm (IQR)	44.3.96 (34.51–55.22)	49.82 (31.03–62.16)	0.203
Average EEM CSA, ${\mathrm{mm}}^{2}$ (IQR)	15.69 (13.13–17.58)	14.87 (12.68–18.03)	0.533
Average lumen CSA, ${\mathrm{mm}}^{2}$ (IQR)	7.91 (6.54–9.32)	8.04 (6.62–10.02)	0.381
Average P+M CSA, ${\mathrm{mm}}^{2}$ (IQR)	8.49 (7.21–10.43)	9.02 (8.55–11.43)	0.109
Plaque burden, %	47.32 (42.76–51.95)	48.21 (43.29–53.21)	0.441
Reference diameter, mm	3.1 ± 0.51	3.18 ± 0.48	0.514
MLD, mm	2.64 ± 1.44	2.41 ± 1.53	0.437
MLA, ${\mathrm{mm}}^{2}$	2.81 ± 0.64	2.93 ± 0.62	0.741
Reference area, ${\mathrm{mm}}^{2}$	13.94 ± 4.61	14.44 ± 5.07	0.578

Note: values are mean ± standard deviation (SD) or median (interquartile range). Abbreviations: EEM, external elastic membrane; CSA, crosssectional area; PSM, propensity score matching; P+M, plaque+media; IVUS, intravascular ultrasound; MLD, minimum lumen diameter; MLA, minimal lumenal area; IQR, interquartile range.

### 3.4 Independent Predictors of MACE

By multivariate analysis, diabetes mellitus (DM) was found to have a positive correlation with MACE, but the existence of CN was found to have a negative association with MACE (Table 6). The presence of CN remained significantly associated with MACE after PSM (Table 7).

**Table 6.** **Multivariate analysis of predictors for nonculprit-lesion MACE**.

Variables	HR	95% CI	*p* value
Age	1.019	0.980–1.058	0.350
Diabetes mellitus	2.216	1.246–3.940	0.007
Prior myocardial infarction	1.304	0.675–2.519	0.430
CKD (eGFR <60)	1.144	0.619–2.113	0.668
Calcified nodule	0.341	0.140–0.829	0.018
MLD	1.075	0.892–1.296	0.449

Abbreviations: MACE, major adverse cardiovascular events; CKD, chronic kidney disease; eGFR, estimated glomerular filtration rate; 95% CI, 95% confidence interval; HR, hazard ratio; MLD, minimum lumen diameter.

**Table 7.** **Multivariate analysis of predictors for nonculprit-lesion MACE after PSM**.

Variables	HR	95% CI	*p* value
Age	1.022	0.964–1.083	0.474
Diabetes	1.665	0.715–3.878	0.238
Prior myocardial infarction	2.063	0.775–5.493	0.147
CKD (eGFR <60)	1.563	0.586–4.167	0.372
Calcified nodule	0.275	0.108–0.703	0.007
MLD	1.272	0.953–1.699	0.103

Abbreviations: MACE, major adverse cardiovascular events; CKD, chronic kidney disease; eGFR, estimated glomerular filtration rate; PSM, propensity score matching; 95% CI, 95% confidence interval; HR, hazard ratio; MLD, minimum lumen diameter.

### 3.5 Clinical Outcomes between Patients with CN and without CN

**Fig. 4.**
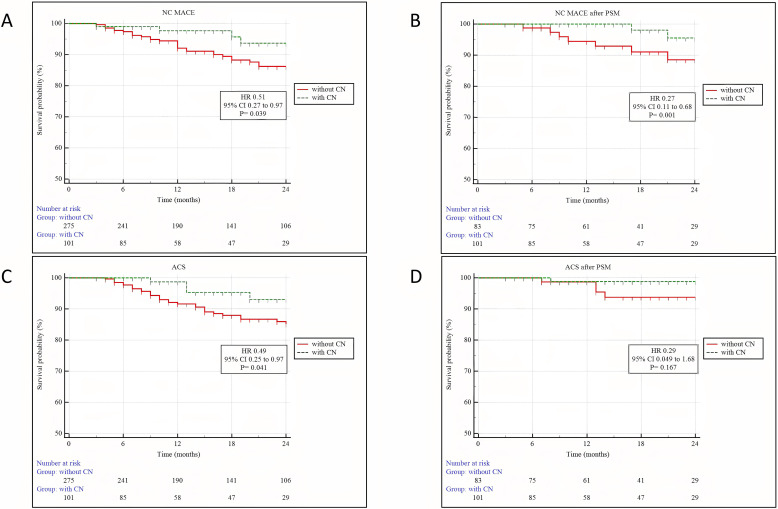
**Kaplan–Meier analysis of cardiac outcomes between patients with and without CN**. (A) NC MACE. (B) NC MACE after PSM. (C) ACS. (D) ACS after PSM. Abbreviations: NC MACE, nonculprit-lesion major adverse cardiovascular events; PSM, propensity score matching; ACS, acute coronary syndrome; 95% CI, 95% confidence interval; HR, hazard ratio; CN, calcified nodules.

Clinical outcomes and hazard ratios adjusted for PSM are summarized in Tables 8,9 and Fig. 4. Over the 19.35 ± 10.59 month observation period, the occurrence of MACE in non-culprit lesions was significantly lower. This difference was primarily driven by the reduced incidence of ACS, while the rates of cardiac-related death and re-hospitalization due to unstable or progressive angina were similar between patients with and without CN. Similarly, a significant difference in the occurrence of non-culprit-lesion MACE was observed after PSM.

**Table 8.** **Nonculprit-lesion MACEs for clinical outcomes**.

Variables	Calcified nodules (n = 101)	Without calcified nodules (n = 275)	*p* value
Nonculprit-lesion MACE	6 (5.9)	46 (16.7)	0.046
Cardiac-cause death	5 (5.0)	7 (2.5)	0.198
Recurrence of ACS	5 (5.0)	40 (14.5)	0.049
Rehospitalization	2 (2.0)	6 (2.2)	0.482

Abbreviations: MACE, major adverse cardiovascular events which included cardiac-cause death, the recurrence of ACS resulting from the progression of non-culprit lesion, or rehospitalization due to unstable or progressive angina. ACS, acute coronary syndrome; 95% CI, 95% confidence interval; HR, hazard ratio.

**Table 9.** **Nonculprit-lesion MACEs for clinical outcomes after PSM**.

Variables	Calcified nodules (n = 101)	Without calcified nodules (n = 83)	*p* value
Nonculprit-lesion MACE	4 (4.0)	15 (18.1)	0.011
Cardiac-cause death	1 (1.0)	1 (1.2)	0.959
Recurrence of ACS	1 (1.0)	4 (4.8)	0.237
Rehospitalization	2 (2.0)	3 (3.6)	0.685

Abbreviations: MACE, major adverse cardiovascular events which included cardiac-cause death, the recurrence of ACS resulting from the progression of non-culprit lesion, or rehospitalization due to unstable or progressive angina. ACS, acute coronary syndrome; 95% CI, 95% confidence interval; HR, hazard ratio; PSM, propensity score matching.

## 4. Discussion

The following are the key conclusions of the current study addressing CN in non-culprit lesions: (1) CN was detected in 16.9% of arteries and in 26.9% of patients with ACS in non-culprit lesions; (2) non-culprit CN was more commonly found in the mid-portion of the RCA and at bifurcation sites; (3) non-culprit CN exhibited a negative association with MACE; (4) patients with non-culprit CN had a significantly lower incidence of MACE.

### 4.1 The Prevalence of CN

Based on pathologic examination, CN, protruding into the lumen exhibited thin fibrous cap derived from disruptive calcified nodules, filled a non- or occlusive platelet/fibrin thrombus [1]. IVUS criteria for calcified nodules showed a luminal surface with irregular, protruding, and convex appearing lesions [11]. Using optical coherence tomography (OCT), CN was defined as a lesion with a disruptive fibrous cap in associated with a calcified plaque, protruding calcium, superficial calcification, and the presence of extensive calcification [13]. Near-infrared spectroscopy (NIRS) findings shown NIRS-CN was associated with convex calcium deposits with a maximum lipid core burden index of 4 mm (${\mathrm{MaxLCBI}}_{\text{4mm}}$) and was 355 [IQR: 303 to 478] [14]. Among them, only 7.3% of all calcified nodules were visible during standard coronary angiography in the present research. In this study, we observed a 26.9% incidence of non-culprit CN in patients with ACS, whereas previous reports have indicated a prevalence of culprit CN ranging from 2.4% to 8% [3, 13, 15, 16]. The evaluation of non-culprit lesions, which were shown to be spread among the three epicardial coronary vessels, could explain the greater incidence seen in our study. Employing OCT, 7.3% of the 3231 patients exhibited either eruptive or noneruptive CN [17]. More recently, CN was discovered to be the underlying morphology of heavily calcified lesions (CLs) in 48.5% (n = 128 out of 264) of patients with high-risk features who required rotational atherectomy [4]. Furthermore, among patients with long-standing hemodialysis and a high burden of calcium, the prevalence of CN was 26% (n = 44 out of 114), with eruptive CN occurring in 26% of all CN lesions [18]. Regarding the pathogenesis of ACS, Sugane *et al*. [19] demonstrated the presence of eruptive calcified masses in 5.3% (n = 35 out of 657) of all ACS patients. Similarly, Wolny *et al*. [20] examined 224 LMCA bifurcations in patients who had undergone previous CABG (n = 78) and those without prior CABG (n = 148), and found a significantly higher prevalence of CN in the LMCA among post-CABG patients compared to those without prior CABG (36.8% vs. 2.7%).

### 4.2 Clinical Presentation of Patients with CN

Advanced age, DM, and other risk factors have been recognized as contributing factors to the development of CA calcification [21, 22]. Additionally, we discovered that age and DM were independently associated with the development of CN in this study, while there was no difference between the two groups in baseline characteristics after PSM. Previous studies concentrated on the clinical features of patients with CN at the culprit lesion. CN has been linked to CABG, and it is thought that changes in vascular shear stress caused by decreased native CA flow may lead to the development of CN [20]. Kobayashi *et al*. [3] found that CN was observed more frequently by OCT in older individuals and those with DM than in those without CN. Additionally, Sugane *et al*. [19] also found that CN patients were more likely to have coronary risk factors such as CKD, continuous hemodialysis, and a history of PCI. Given that decreased kidney function can result in abnormal calcium and phosphorus metabolism, as well as the release of calcification-related proteins and inflammatory cytokines, these CKD-related variables could play a major role in the development of CN [23].

### 4.3 Location of CN

There is a scarcity of information on the distribution of non-culprit CN. However, our observations indicate that the mid segment of the RCA is more frequently affected by non-culprit CNs. Mechanical stress caused by coronary hinge motion during cardiac pulsations may lead to the formation of eruptive CN, and the mid segment of the RCA is more vulnerable to this impact caused by cardiac motion [1]. The axial position of non-culprit CN was investigated in 185 patients in a recent study [24], which demonstrated that non-culprit CNs were predominantly located in the proximal segments of the LAD and LCx. Similarly, in an OCT study [17] involving 3231 consecutive patients, the location of eruptive or noneruptive CNs was analyzed, revealing a tendency for CNs to cluster within the proximal segments of the LAD and the proximal to mid segments of the RCA. Another OCT study by Lee *et al*. [16] reported a similar finding, with CN tending to cluster in the ostium or mid portion of the RCA. Interestingly, in our study, CN were predominantly located at the bifurcating regions of coronary arteries. Notably, in patients with CABG, 85.7% of CNs were found within 5 mm to the LMCA bifurcation, which could potentially impact the delivery and expansion of balloons and stents [20]. Pathology investigations have thoroughly detailed the spatial distribution of atherosclerosis within a coronary bifurcation, finding atherosclerotic plaque formation on the lateral walls but largely undamaged flow [25]. CN lesions with fibrous layer elements have the highest degree of calcification relative to plaque area of any vulnerable plaque subtype and are thought to be related with healed fibroatheromas [26]. Based on these concepts, we can speculate on the mechanisms underlying CN development in bifurcating regions.

### 4.4 Clinical Outcomes in CN

In the age of drug-eluting stents (DES), severely CLs with CN are anticipated to have a negative impact on PCI results [4]. Target lesion revascularization (TLR) is more frequently required following PCI of lesions with severe calcification compared to those without [27]. Previous research has found TLR rates ranging from 20.0% to 38.0% after 2 years, mainly in unselected CLs and especially in the group with eruptive CN [3, 4, 28, 29]. Morofuji *et al*. [4] discovered that CN was present in half of the severely CLs that required rotational atherectomy, and that CN was related with worse adverse outcomes after a 5-year follow-up period. The recurrence of CN within the implanted DES was responsible for more than 80% of TLR at the CN lesion [19]. A recent OCT study found a 2-year cumulative rate of target lesion failure (TLF) caused primarily by clinically induced TLR. This study indicated that eruptive CN morphology has a different influence on long-term clinical outcomes when compared to non-eruptive CN morphology [17]. Recently, a previous OCT study [30] demonstrated that patients with eruptive CN had a remarkably higher 2-year incidence of cumulative MACE compared with the calcified protrusion and superficial calcific sheet groups. This finding suggest that eruptive CNs in culprit lesions with ACS patients are more frequently to impact clinical adverse outcomes after PCI. However, it remains unclear whether CN in non-culprit lesions with ACS patients is considered as the reason for adverse outcomes. There have been no previous systematic studies using intracoronary imaging modalities that have shown an influence of CN in non-culprit lesions. Xu *et al*. [24] found that CN in non-culprit lesions of ACS patients resulted in better clinical outcomes over a 3-year follow-up period, which is consistent with our current investigation. Surprisingly, no deaths, cardiac arrests, or myocardial infarctions occurred in the CN group. While one pathology group has described culprit CN as a rare cause of coronary thrombosis [1], non-culprit CN may represent precursor lesions similar to thin-cap fibroatheromas (TCFs). It is important to note that CNs do not always cause thrombosis, and TCFs do not always cause plaque rupture (PR). In the current study, we found the CN group have less non-culprit lesion MACEs compared with the non-CN group. We hypothesize that CNs could be the result of plaque rupture (PR), thrombosis, and subsequent healing rather than the cause of poor outcomes. Furthermore, CNs in non-culprit lesions may help to stabilize the lesion rather than being the cause of adverse events.

## 5. Limitations

This study has several limitations. First, it was a retrospective single-center study, which may have introduced an element of selection bias. Additionally, the number of lesions with CN was relatively small, limiting the generalizability of the findings. Another limitation is the lack of pathological assessments for non-culprit lesions with and without CN. Although IVUS is commonly used for evaluating coronary calcification, its resolution may not be sufficient to visualize small nodular calcifications. Furthermore, it is important to consider that intensive medical care in compliant patients may have mitigated the occurrence of MACE during the follow-up period, potentially influencing the study outcomes. Therefore, a larger, prospective, randomized study is needed to further investigate and validate our findings.

## 6. Conclusions

The occurrence of CN at non-culprit lesions in patients with ACS was prevalent and caused fewer adverse clinical outcomes.

## Data Availability

The datasets generated during and/or analyzed during the current study are available from the corresponding author on reasonable request.

## Abbreviations

CN, calcified nodule; ACS, acute coronary syndrome; IVUS, intravascular ultrasound; PSM, propensity score matching; MACE, major adverse cardiovascular event; CI, confidence intervals; HR, hazard ratio; PCI, percutaneous coronary intervention; TLR, target lesion revascularization; IRS, refractory in-stent restenosis; STEMI, ST-segment elevation myocardial infarction; NSTEMI, nonST-segment elevation myocardial infarction; uAP, unstable angina pectoris; MLA, minimum lumen area; QCA, quantitative coronary angiography; LM, left main; MLA, minimum lumen area; EEM, external elastic membrane; CSA, cross-sectional area; PB, plaque burden; IQRs, interquartile ranges; CABG, coronary artery bypass grafting; OCT, optical coherence tomography; CKD, chronic kidney disease.
